# A Study on Biofouling and Cleaning of Anion Exchange Membranes for Reverse Electrodialysis

**DOI:** 10.3390/membranes12070697

**Published:** 2022-07-08

**Authors:** Gonçalo Tiago, Maria Beatriz Cristóvão, Ana Paula Marques, Rosa Huertas, Ivan Merino-Garcia, Vanessa Jorge Pereira, João Goulão Crespo, Svetlozar Velizarov

**Affiliations:** 1 LAQV-REQUIMTE, Department of Chemistry, NOVA School of Science and Technology, Universidade NOVA de Lisboa, 2829-516 Caparica, Portugal; g.tiago@campus.fct.unl.pt (G.T.); b.cristovao@ibet.pt (M.B.C.); rosa.maria.huertaspenela@gmail.com (R.H.); jgc@fct.unl.pt (J.G.C.); 2 IBET-Instituto de Biologia Experimental e Tecnológica, Apartado 12, 2780-901 Oeiras, Portugal; ana.marques@ibet.pt (A.P.M.); vanessap@ibet.pt (V.J.P.); 3 Departamento de Ingenierías Química y Biomolecular, Universidad de Cantabria, Avda. Los Castros, s/n, 39005 Santander, Spain; ivan.merino@unican.es; 4 Instituto de Tecnologia Química e Biológica António Xavier, Universidade Nova de Lisboa, Av. da República, 2780-157 Oeiras, Portugal

**Keywords:** anion exchange membranes, reverse electrodialysis, biofouling, membrane cleaning

## Abstract

This study covers the modification, (bio)fouling characterization, use, and cleaning of commercial heterogeneous anion exchange membranes (AEMs) to evaluate their feasibility for reverse electrodialysis (RED) applications. A surface modification with poly (acrylic) acid resulted in an improved monovalent perm-selectivity (decreased sulfate membrane transport rate). Moreover, we evaluated the (bio)fouling potential of the membrane using sodium dodecyl sulfate (SDS), sodium dodecyl benzenesulfonate (SDBS), and *Aeromonas* *hydrophila* as model organic foulants and a biofoulant, respectively. A detailed characterization of the AEMs (water contact angle, ion exchange capacity (IEC), scanning electron microscopy (SEM), cyclic voltammetry (CV), and Fourier Transform Infrared (FTIR) spectra) was carried out, verifying that the presence of such foulants reduces IEC and the maximum current obtained by CV. However, only SDS and SDBS affected the contact angle values. Cleaning of the biofouled membranes using a sodium hypochlorite aqueous solution allows for (partially) recovering their initial properties. Furthermore, this work includes a fouling characterization using real surface and sea water matrixes, confirming the presence of several types of fouling microorganisms in natural streams. A lower adhesion of microorganisms (measured in terms of total bacteria counts) was observed for the modified membranes compared to the unmodified ones. Finally, we propose a cleaning strategy to mitigate biofouling in AEMs that could be easily applied in RED systems for an enhanced long-term process performance.

## 1. Introduction

Nowadays, the global energy crisis is one of the most important issues to address, as the worldwide energy consumption is continuously increasing. However, the major problem is that the fossil fuels used for generating energy are finite, non-renewable, and environmentally unfriendly. Although renewable sources such as wind and solar energies represent a good alternative, they are not enough to satisfy the increasing energy demand, which is expected to grow by 70% by 2050 [1]. A potential approach is “blue” energy harvesting by bringing into contact two natural water streams with different salinity, the so-called salinity gradient power (SGP) technology [2]. Among the different available technologies, reverse electrodialysis (RED) could be used to generate electricity from natural salinity gradients such as the one between seawater and river water. A life cycle assessment of SGP has recently shown fewer negative impacts for the production of the same amount of power and lower sensibility to seasonal variations compared to alternative renewable energy-harvesting processes [3].

In recent years, significant progress has been made in the RED technology focusing on its optimization before large-scale implementation. The RED performance has been optimized in terms of numerous operating variables including, among others, (i) membrane properties, (ii) compartment and spacer design, (iii) saline streams concentrations defining salinity gradient, and (iv) flow velocity and hydrodynamics [4,5,6].

Nevertheless, one of the main remaining challenges that RED faces before becoming practically feasible at a larger scale is the development of (bio)fouling on the ion exchange membranes (IEMs) involved in the system, thus decreasing the obtainable net power output [7]. Among IEMs, anion exchange membranes (AEMs) are much more prone to organic fouling due to the negative charge of natural organic matter (NOM) compounds present in natural aqueous streams, which are easily attracted to their positively charged surface [8].

As a consequence, different surface modification procedures have been proposed to mitigate the impact of (bio)fouling of AEMs, and also to enhance their monovalent ion permselectivity [9]. For instance, Guler et al. prepared AEMs by amination, reaching a power density of 1.27 W/m^2^. The authors performed an amination/crosslinking of polyepichlorohydrin (PECH) polymers, and added 4-diazabicyclo [2.2.2]octane (DABCO) to introduce positive charges [10].

Antifouling membranes were fabricated by surface modification of a commercial Fujifilm type I AEM with zwitterionic layers by Pintosi et al. [11]. The antifouling behavior of the membranes in RED was evaluated using artificial river and seawater and sodium dodecyl benzenesulfonate (SDBS) as the model foulant. The modified membranes were found to be effective in delaying the fouling onset.

Güler et al. used 2-acryloylamido-2-methylpropanesulfonic acid as a modifying agent, resulting in AEMs with a negatively charged coating, which showed increased hydrophilicity and exhibited sufficient antifouling potential against organic foulants [12]. Another approach was developed by Hong and Park using a mixture of poly (diallyldimethylammonium chloride) (PDDA) and polyvinyl alcohol (PVA) to prepare AEMs, which were tested in a lab-scale RED stack [13]. The highest gross power density of 0.58 W/m^2^ was obtained at a PDDA/PVA blend ratio of 1.5. Nagarale et al. reported that it is possible to prepare an AEM with antifouling properties by coating and curing a thinner hydrophilic urethane acrylate layer on its surface without scarifying its electrochemical characteristics [14].

Other researchers have used PVA as a green modifying AEM surface agent, by cross-linking it with a dye containing negatively charged sulphonic groups, thereby decreasing the tendency of negatively charged compounds to adsorb on the positively charged AEM [15]. The reason for the usage of PVA is mainly related to its low chemical resistance and high antifouling potential [16]. Moreover, poly (styrenesulfonate) (PSS) has also been proposed to modify commercial AEM membranes for RED applications, achieving good monovalent permselective properties and antifouling behavior [17].

In our previous work, we found that poly (acrylic) acid (PAA) can also be an appropriate (green and cheap) surface modifying agent for AEMs [18]. The membranes after PAA modification presented a higher hydrophilicity and an improved monovalent ion perm-selectivity compared to the pristine ones. The main reason that supports the use of PAA as a modifying agent is its eco-friendly behavior compared to other alternative modifying agents. For example, its lethal dose (LD50) is higher in comparison with alternative chemicals used such as polyethylenimine (PEI) and poly (dopamine) (PDA), among others, as illustrated in Figure S1 (please see the Supplementary Materials). The higher the LD50 value, the lower the toxicity of a given chemical.

Besides fouling by organic compounds, it is worth mentioning that biofilms can also grow over the surface of AEMs during their exposure to natural feedwaters, thus hampering the overall performance of RED for blue energy harvesting. It was recently demonstrated in a 54-day-long study with natural feedwaters that the presence of biopolymers caused a reduction in gross power density [19]. Therefore, deep knowledge on the causes of biofouling and the development of adequate strategies for its mitigation are highly required [20]. A recent study reported AEMs with increased resistance to biofouling by modifying them with PDA, reaching up to a 40 % reduction in biomass accumulation [21]. Bacteria-mediated biofouling is caused by the attachment of planktonic bacteria, followed by the proliferation of sessile colonies on the membrane surface [22]. Herzberg et al. demonstrated biofilm formation of *Pseudomonas aeruginosa* on AEMs, causing a reduction in the transport rates of anions that was more pronounced for heterogeneous AEMs than for homogeneous ones [23].

A particular case of biofouling is that of *Aeromonas*. These bacteria are Gram-negative, facultative anaerobic, oxidase- and catalase-positive, fermentative, and mostly motile bacilli. *Aeromonas* are present in several aquatic environments such as seawater, irrigation water, river water, brackish water, freshwater, groundwater, spring water, sewage-contaminated water, and activated sludge, among others [24,25]. *Aeromonas* are considered as environmental opportunistic pathogens for both animals and humans, and can be responsible for furunculosis and septicemia in fish [26]. In humans, they can cause gastroenteritis, wound infections, bacteremia, and, less frequently, respiratory infections, hepatobiliary infections, peritonitis, urinary tract infections, and ocular infections [27]. The members of *Aeromonas* are characterized by a remarkable ability to colonize a wide range of habitats. Typically, many of its colonization aspects rely on biofilm production and cell-to-cell signaling. *Aeromonas* within a biofilm are more resistant to disinfectants than planktonic cells, as shown for *Aeromonas hydrophila* strains [28]. These bacteria have thus been recovered from biofilm in drinking water distribution systems [24,25], even when water supply is chlorinated. They have been described as avid biofilm formers as well as due to their numerous virulence mechanisms [29]. Their presence was also found to be higher than—and, in some cases, correlated with—fecal coliforms (classical indicators of water fecal pollution) [30]. Importantly, they have been reported as prevalent in river water and seawater with different degrees of pollution, thus making them most relevant for real RED process operations installed at such locations. Therefore, *Aeromonas hydrophila* was selected as a target biofouling species in the present study.

Due to the negative effects of (bio)fouling on ion exchange membrane process performance in general, various cleaning approaches have been reported in the literature [31]. Although most of the cleaning strategies have been developed for electrodialysis (ED) applications, these could be easily adapted for RED systems. A cleaning strategy proposed by Barros et al. [32] in the treatment of electroplating industry effluents through ED involved the use of alkaline (NaOH) solutions of different concentrations. The results showed that lower/milder NaOH concentrations are more effective in recovering membrane performance for this application. A comparison of the efficacy of acidic (HCl) and alkaline (NaOH) solutions has also been realized for the treatment of oily wastewater streams by ED systems [33]. The acidic cleaning was found to be more effective than the alkaline one in that case. Bdiri et al. demonstrated that ethanol and seawater can provide a significant recovery of the ion exchange capacity (IEC) and membrane conductivity [34,35]. A combination of acidic (HCl) and alkaline (NaOH) cleaning methods, as well as the use of the surfactant SDBS has been recently applied in the treatment of ion exchange membranes used in ED for the desalination of polymer flooding produced water [36]. The results showed the importance of acidic and alkaline cleaning methods for the removal of polyacrylamide-based foulants. The surfactant sodium dodecyl sulfate (SDS) was used as an AEM model foulant by Zhao et al., where a mild cleaning treatment procedure involving ultrapure water was adopted [37]. The cleaning effect of water was comparable to that of acidic cleaning, while the alkaline one was less effective. The effect of using strong oxidizing agents (such as sodium hypochlorite) has been previously investigated for the cleaning of fouled ion exchange membranes used in ED [38] and RED [39], respectively.

In the present study, we focused on investigating the effect of a biofoulant (*Aeromonas)* and two model organic foulants (SDS and SDBS) on the properties of pristine (unmodified) and PAA-modified heterogenous anion exchange membranes, previously reported by Merino-Garcia et al. [18]. The effects of chemical cleaning of the fouled membranes with sodium hydroxide and sodium hypochlorite-based solutions were assessed and compared.

## 2. Materials and Methods

### 2.1. Chemicals and Membranes

Commercial polyester-based heterogeneous Ralex AEMs were purchased from MemBrain s.r.o. (Stráž pod Ralskem, Czech Republic). Sodium chloride (NaCl, Applichem PANREAC (Barcelona, Spain)), 99.8% was used to simulate river water and seawater. Sodium sulfate (Na_2_SO_4_) anhydrous and sodium dodecyl sulfate (SDS) were purchased from Applichem PANREAC (Barcelona, Spain). Sodium dodecyl benzenesulfonate (SDBS) and poly (acrylic) acid (PAA), the latter used for membrane modification, Trizma^®^ hydrochloride ≥99.0% and Trizma^®^ base ≥99.9% were purchased from Sigma-Aldrich (St. Louis, MO, USA). Potassium chromate (K_2_CrO_4_) 99.5% was purchased from Acros Organics (Geel, Germany), and silver nitrate (AgNO_3_), 99+% was acquired from Alfa Aesar (Ward Hill, MA, USA).

### 2.2. Surface Membrane Modification

The monolayer surface modification procedure followed in this study was adapted from the work developed by Merino-Garcia et al. [18]. Briefly, a 0.1 M Trizma buffer solution was prepared by dissolving 14.04 g Trizma HCl and 1.34 g Trizma base in 1 L of deionized water. Then, 300 mg of PAA was dissolved in 100 mL of the previously prepared Trizma solution, resulting in 3 g PAA/L as the optimal concentration found in our previous study [18]. The AEM was then immersed in the modifying solution for 24 h at room temperature. Afterwards, the modifying solution was replaced by a Trizma buffer solution for membrane washing. The membranes were finally immersed in deionized water until use.

### 2.3. Membrane Surface Characterization

#### 2.3.1. Contact Angle

The water contact angle of a sessile drop was determined to evaluate the surface hydrophilicity of the membranes using DSA25B equipment (KRUSS technology, Hamburg, Germany), which is fully computer-controlled by automatic image-based analysis. For this test, several pieces of flat membranes (9 cm^2^ each) were used after washing with deionized water and drying overnight at 40 °C. A sessile drop of distilled water with a volume of 2 μL was put onto the membrane surface using a syringe with a diameter of 0.6 mm, while a camera captured the drop over time (consecutive frames). Ten frames were attained for each measurement, with a frame interval of 100 ms. The contact angle was measured on at least three random positions, and then the average values were reported as a final result, taking into account the standard deviation.

#### 2.3.2. Fourier Transform Infrared Spectroscopy

Fourier Transform Infrared Spectroscopy (FTIR), using the attenuated total reflectance (ATR) mode, was used to evaluate the chemical structure of modified and unmodified membrane samples. The FTIR analyses were performed using a Bruker Spectrometer IFS 66/S FT-IR instrument (Bruker Corporation, Billerica, MA, USA) equipped by H-ATR with ZnSe crystal. Prior to analysis, the membrane samples were dried in a desiccator overnight at a room temperature. The analysis was performed in 3 random membrane positions to check the reproducibility of the response. The spectra were obtained in the 4000–550 cm^−1^ range over 220 scans, with 2 cm^−1^ resolution.

#### 2.3.3. Scanning Electron Microscopy (SEM)

The morphology of the top and cross-sections of the membrane samples was characterized by scanning electron microscopy (SEM) images. The samples were prepared by sputtering with an Au/Pd thin film using a Quorum Technologies Q150T ES model apparatus and analyzed using a JSM 7001F SEM microscope (JEOL USA Inc., Peabody, MA, USA).

### 2.4. Static Assays

#### 2.4.1. Fouling Experiments

##### Water Sampling and Characterization

Surface and sea water samples were collected in clean, sterilized 2 L glass bottles from Tagus River and the Atlantic Ocean, respectively. All samples were transported to our laboratory facilities and kept at 4 °C until use. A physical and chemical characterization was performed by measuring pH (Micro pH 2002, Crison, Barcelona, Spain), total solids, and total suspended solids (TSS) (using the standard method 2540), as well as chemical oxygen demand (with Hach Lange HT200S (Loveland, CO, USA), Hach LangeDR2800 apparatus, and the LCK 1814 kit).

In addition, all samples were subjected to a microbiological characterization. A total viable bacterial count was performed according to the general standard methods for examination of water (ISO 6222:1999). Briefly, 100 mL of water sample was filtered through a 0.22 µm porous membrane, placed on agar plates, and incubated at 30 °C for 24 h. Total coliforms and *E. coli* were also determined using enzyme-specific rapid microbial methods, Colilert^®^-18/Quanti-Tray^®^ from IDEXX, and described on standard ISO 9308-2:2012.

##### Aeromonas Isolation and Identification

To isolate Aeromonas hydrophila, 100 mL of river water (Tagus River, Algés, Portugal) was filtered through a 0.22 µm nitrocellulose membrane (Merck Millipore, Burlington, MA, USA). After filtration, the membrane filter was transferred to a growth medium and incubated at 30 °C for 24–36 h. Colonies of different morphologies were selected and streaked on tryptic soy agar (TSA) plates at 30 °C for 24 h to purify the different colonies. This step was repeated two more times to guarantee purified colonies. After this purification procedure, an isolated colony was randomly selected and resuspended on 10 µL of sterile phosphate buffer solution (1X solution, pH 7.4) and applied to Whatman^®^ FTA^®^ Cards (Merck KGaA, Darmstadt, Germany). Subsequently, a dried sample was identified by 16S rRNA gene sequencing at STAB Vida (Costa de Caparica, Portugal) sequencing services. The isolated strain was identified as A. hydrophila. This isolate, which presented 99.93% of homology with A. hydrophila strain T50-2 (NCBI accession number MK656384.1), was used in the biofouling experiments.

##### Membrane Biofouling

Coupons of unmodified and modified membranes were immersed in 500 mL of river and seawater samples for 14 days. In parallel, samples of modified and unmodified membranes were immersed in 100 mL cell suspension of A. hydrophila (10^8^ CFU/mL). Control samples of modified and unmodified membranes were immersed in Milli-Q water and exposed to the same conditions. All the vials were continuously shaken at 120 rpm and kept at 30 °C. After 14 days, duplicate membrane samples and controls were taken and stored in 0.1 M NaCl to prevent the alteration of their fouling potential. The surface of the membranes was characterized by performing contact angle measurements, FTIR, and SEM analyses. Detailed biological analyses were also performed to evaluate the level of microorganisms adsorbed to the membranes. Briefly, after 14 days of immersion of the modified and unmodified membranes in surface and sea water as well as in the Aeromonas cell suspension, the small membrane coupons were placed in a sterile container with 5 mL of a sterile phosphate-buffered saline solution. After vortex mixing twice, 100 µL of sample were spread in a petri dish with TSA and incubated at 37 °C for 24 h. For analysis of total coliforms and *E. coli*, samples were placed in a Quanti-Tray and incubated at 35 °C for 18 h.

The SEM analyses of the Aeromonas test involved an additional treatment, previously described by Oliveira et al. [40].

#### 2.4.2. Effect of Chemical Cleaning on Membrane Surface

Regarding sodium hydroxide, different concentrations of NaOH were tested, including a low concentration level (0.1 mol/L), as this concentration was found to be most effective in restoring the original properties of anion exchange membranes (HDX200) used in the treatment of synthetic wastewater from the electroplating industry [32], while the highest concentrations used (0.5 and 1.0 mol/L) seemed to degrade the membranes. According to the recommendation provided by the manufacturer of the Ralex AEMs, the highest NaOH concentration tested in this study was set at 30 g/L. With respect to the sodium hypochlorite cleaning solution, a concentration of 3 mg/L as total free chlorine was considered based on previous literature [41].

Briefly, duplicate coupons of fouled membranes (2 cm × 1 cm) were immersed in 250 mL of sodium hydroxide or sodium hypochlorite solutions in sealed glass vials for 7 days. The sodium hypochlorite solution was renewed every 48 h. All glass vials were continuously shaken at room temperature (T = 21 °C) and covered with aluminum foil to avoid the degradation of the cleaning solutions. In addition, control samples of unmodified and modified membranes were immersed in Milli-Q water and exposed to the same conditions. After 7 days, all the membrane samples were collected and rinsed with Milli-Q Water. Afterwards, they were analyzed via FTIR, SEM, and contact angle measurements. Prior to characterization, all samples were kept in a 0.1 M NaCl solution.

### 2.5. Ion Exchange Capacity

The ion exchange capacity (IEC) was determined according to the Mohr titration method. First, wet membranes were immersed in 0.4 M NaCl for 24 h. After that, this solution was replaced by 0.2 M Na_2_SO_4_, in which the membranes were immersed for 3 h, allowing the complete replacement of Cl^−^ by SO_4_^2−^. The resulting solution was titrated with a 0.1 M AgNO_3_ solution to estimate the released Cl^−^ amount. In each measurement, a few drops of 0.1 M K_2_CrO_4_ (indicator) were added. The titration finished when K_2_CrO_4_ changed its color from yellow to red. The membranes were then dried in order to obtain their dry masses.

### 2.6. Electrochemical Measurements and Sulfate Mass Transport Experiments

These experiments were carried out in a two-compartment diffusion cell, described in detail elsewhere [18], in order to evaluate the membrane conductivity and the sulfate transport rate through unmodified and modified membranes. In sulfate mass transport experiments, simulated synthetic river water (1 g/L NaCl + 0.1 g/L Na_2_SO_4_) was used. Seawater mimicked by using 30 g/L NaCl aqueous solution was placed in the sulfate-receiving compartment. The concentrations of sulphate and sodium ions from the samples withdrawn periodically were estimated based on the determined contents of S and Na elements, measured by inductively coupled plasma–atomic emission spectrometry (ICP-AES) (Ultima model, Horiba Jobin-Yvon, Longjumeau, France) equipped with a radio frequency (RF) generator of 40.68 MHz, a Czerny-Tner type monochromator with 1.00 m (sequential), and a Hydride Generator with a concomitant metals analyzer (CMA), AS500 auto sampler, and data acquisition software. The data obtained can be found in the Supplementary Materials (Figure S8).

The same solutions and the same cell were used to perform cyclic voltammetry measurements using two silver electrodes placed close to each membrane side. The voltammograms were obtained between −0.6 and +0.6 V (3 scans) at a scan rate of 200 mV/s, using an Ivium potentionstat/galvanostat (Ivium Technologies, Eindhoven, The Netherlands). Since the shapes of the voltammograms were very similar, which made it difficult to distinguish between different systems, only the currents obtained at maximum voltage (+0.6 V) are presented, in order to more easily compare the fouling influence via the maximal obtainable currents.

## 3. Results and Discussion

First, the (bio)fouling effects of the membranes surface (including *Aeromonas*) using natural streams are presented and discussed (Section 3.1). It should be noted that the use of real water matrixes is essential to get closer to practical RED applications, which denotes the relevance of this work. Then, the characterization of the membranes in artificial systems (mimicking river water and seawater salinities) is presented (Section 3.2).

### 3.1. Static Assays

#### 3.1.1. (Bio)fouling Experiments

Static (bio)fouling experiments were conducted by immersing modified and unmodified AEMs in surface and sea water (the obtained compositions data are shown in Table 1), as well as in a cell suspension (1.2 × 10^8^ CFU/mL) of *A. hydrophila* isolated from surface water. This isolate was selected due to its reported presence in several aquatic environments and association with biofilm formation [24,25,28]. After 14 days, the target microorganisms (total bacteria count, total coliforms, *E. coli*, and *A. hydrophila*) were quantified on the surfaces of the membranes (Table 2 and Table 3). The data of FT-IR and SEM images for the unmodified and modified membranes can be seen in Figure S2 and Figure S3, respectively, in the Supplementary Materials.

As can be seen in Table 2 and Table 3, a lower adhesion of microorganisms was observed for the modified membranes compared to that of the unmodified membranes, thus demonstrating that the surface modification with PAA conferred anti-adhesion properties to the membranes (an enhanced response against biofouling). This effect is especially notorious for the case of seawater (Table 2). This is in accordance with the work developed by Gratzl et al. [42], who reported that PAA-containing copolymer films displayed an antimicrobial activity against *S. aureus*, *E. coli*, and *P. aeruginosa*, and that the antimicrobial activity increased with increasing acrylic acid content, regardless of the copolymer partner, chain length, and nanostructure.

#### 3.1.2. Effect of the Type of Cleaning Solution

Coupons of modified and unmodified membranes (2 cm × 1 cm, Ralex-AEM each) were immersed in solutions of NaOH (4 g/L and 30 g/L) and a solution of NaClO (3 mg/L), respectively. After 7 days, the membranes were analyzed via FTIR and SEM. The data obtained are displayed in Figure S4 and Figure S5, respectively (please see the Supplementary Materials).

Notorious differences between the unmodified and modified membranes after 7 days of exposure to NaOH and NaClO were not observed, thus demonstrating that any of the cleaning solutions chosen can be used at the concentration levels tested to clean the fouled membranes without compromising the chemical composition and the structure of the membrane samples.

### 3.2. Experiments Using Model Saline Solutions

#### 3.2.1. Hydrophilicity

**Figure 1:**
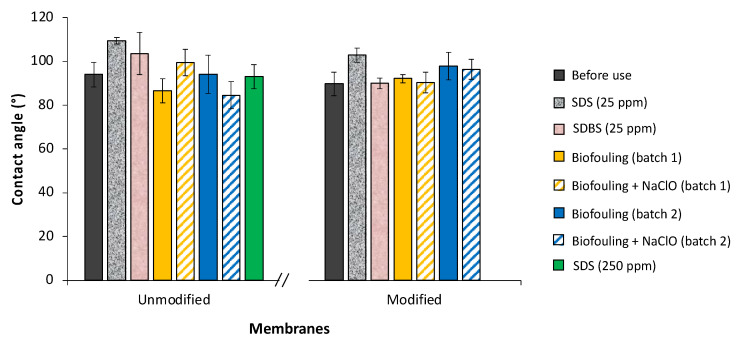
Contact angle results of different (bio)fouled AEMs.

The hydrophilicity of the AEMs was evaluated by water contact angle measurements. Figure 1 shows a comparison of the contact angles’ values of unmodified and modified AEMs with and without fouling using two model chemical foulants: sodium dodecyl sulfate (SDS) and sodium dodecylbenzenesulfonate (SDBS). As can be seen, the PAA-modified membranes showed decreased water contact angle values, except in the presence of both foulants.

Our results are in line with those reported by Zhao et al., who demonstrated the feasibility of layer-by-layer (LbL) assembly of poly (sodium 4-styrene sulfonate) (PSS)/poly (diallyldimethylammonium chloride) (PDADMAC) polyelectrolyte multilayers on a commercial Neosepta AMX membrane [43]. The deposition of a final PSS layer as the top surface facing the bulk solution endowed the surface with negative charges and therefore increased the surface hydrophilicity, leading to an improved antifouling performance to SDS in an ED operation.

The anti-fouling performance of PDA-PSS/TiO_2_ coated AEMs was evaluated with SDBS as a model foulant by Mao et al. [44], who also reported decreased water contact angles for the modified membranes and concluded that the electrostatic repulsion was the main effect enhancing the antifouling ability of the modified membranes.

Remarkably, the increase in contact angle values for the modified AEMs due to the presence of *Aeromonas* can be slightly reduced after membrane cleaning using NaClO, decreasing 2% and 1% for the first batch and for the second batch, respectively.

#### 3.2.2. Ion Exchange Capacity

**Figure 2:**
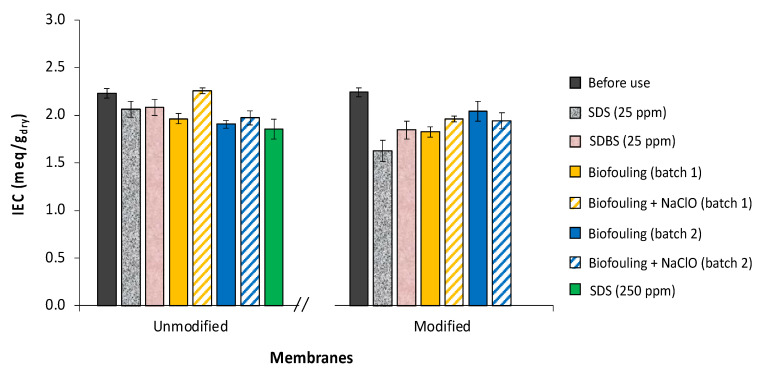
Ion exchange capacity of (bio)fouled membranes in different scenarios.

The IEC was determined for both membranes (modified and unmodified) with and without fouling. Figure 2 shows that for all membranes, the presence of any of the studied chemical foulants (SDS or SDBS) causes a drop in the IEC values when compared to those of the unfouled membranes, thus decreasing their performance. Our results are in agreement with those reported by Lee et al. [45], who investigated the effect of SDBS presence on the ion exchange capacity of Neosepta^®^ AMX membranes, which dropped from 2.5 mmol/g-dried for the unfouled to 2.1 mmol/g-dried for the fouled membrane.

When the membranes were fouled with *Aeromonas*, the IEC values also suffered a drop. For the unmodified AEM, the effect of this biofoulant was more significant than that of SDS or SDBS, whereas for the modified AEM, only SDS had a greater significance on this value than that the biofoulant. Moreover, when the amount of SDS was increased by 10 times for the case of unmodified membranes, this expectedly led to the highest observed drop in IEC.

#### 3.2.3. ATR-FTIR Measurements

**Figure 3:**
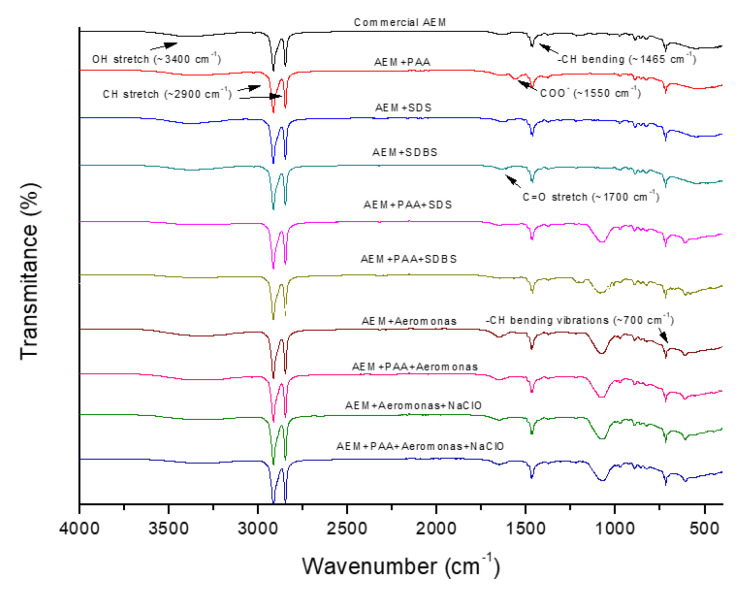
ATR-FTIR spectra of the AEMs. The results of the membranes with *Aeromonas* are from the first batch.

Figure 3 presents a comparison between the spectra obtained for both membranes (modified and unmodified) with and without fouling. For both cases, no significant differences can be found in the basic AEM surface chemical structure. However, the presence of a band at 1550 cm^−1^, which represents the COO^−^ stretch of PAA, confirmed the reproducibility of the modification procedure developed by Merino-Garcia et al. [18] with PAA as an effective modifying agent. Moreover, the presence of any of the model foulants causes the appearance of a new and broad wave at ~1050 cm^−1^, which could be attributed to chemical or physical interactions between the modified membrane surface and the foulant. It is also possible to notice that the presence of foulants on the modified membrane causes a weakening of COO^−^ stretch of PAA. It is worth noting that 1 h exposure to the NaClO solution had no effect on the chemical composition of the membrane, which suggests that it can be used for membrane cleaning.

#### 3.2.4. Electrochemical Measurements

The currents registered at the maximum applied potential (+0.6 V) in the CV analyses were compared for the three different scenarios (unmodified, modified, and fouled AEMs). The results obtained are presented in Table 4.

The presence of PAA at the membranes surface caused an increase in the obtained current, which means an increase in the membrane conductivity. The comparison between the unfouled and the fouled AEMs shows, as expected, that there was a drop in the maximum obtained current when the membranes were previously (bio)fouled (either with SDS/SDBS or with Aeromonas). An explanation for this fact is the partial deposition of SDS, SDBS, or Aeromonas onto the membrane surface, thus reducing the efficacy in transporting charge carriers. A similar observation was verified by Zhao and coworkers, who performed electrodialysis studies using SDS as a model foulant, and verified that the membranes’ conductivity decreases as the SDS concentration increases [37].

During chemical cleaning with NaClO solution in batch 1, the unmodified membranes fouled with Aeromonas partially recovered their conductive properties, whereas the modified ones had an insignificant recovery in this parameter. On the other hand, for batch 2, the membrane recovered its initial current completely, which demonstrates the antibiofouling potential of the prepared membranes.

#### 3.2.5. SEM Analyses

**Figure 4:**
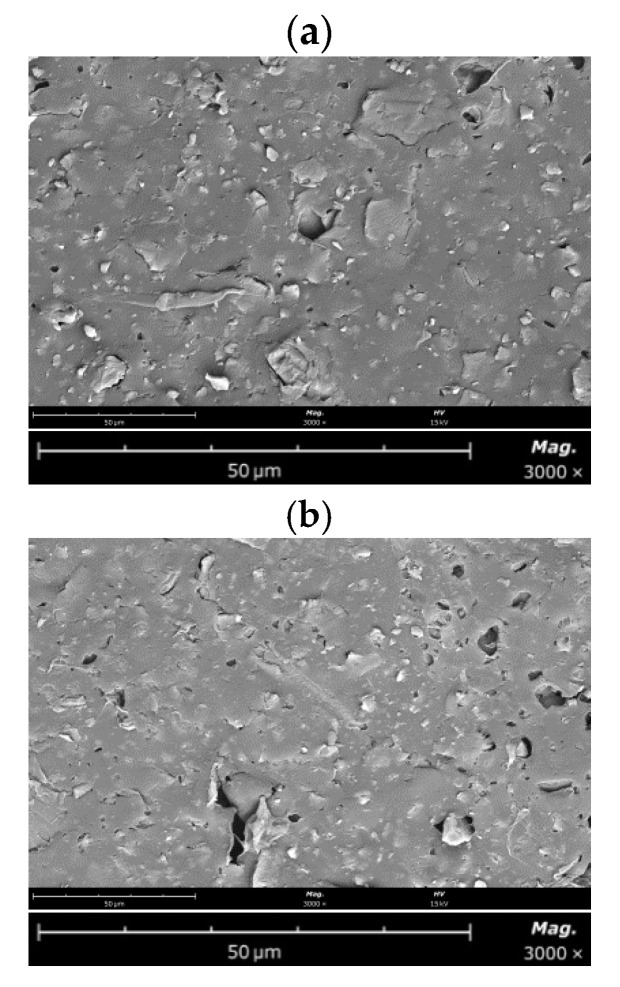
Images of the top surface of the membranes (before biofouling) obtained by SEM: (**a**) unmodified AEM; (**b**) modified AEM.

**Figure 5:**
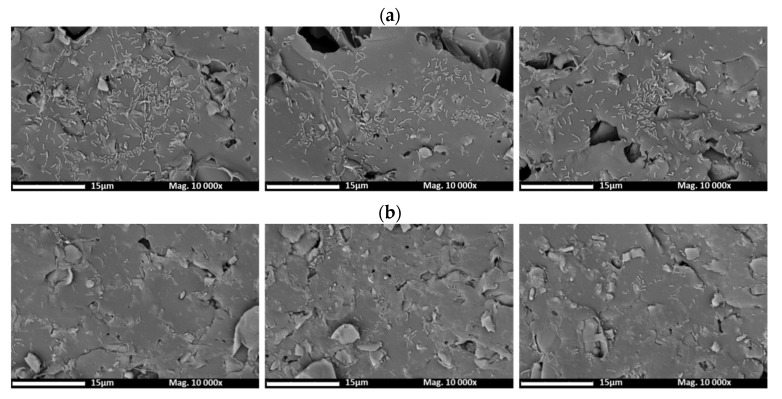
Images of the top surface of the membranes (taken in three different zones) obtained by SEM: (**a**) unmodified membrane with *Aeromonas*; (**b**) modified membrane with *Aeromonas*.

To complement the biofouling analyses, SEM images were taken before (Figure 4) and after (Figure 5) biofouling for the unmodified and modified AEMs.

As it can be observed in Figure 4, the surface structure of both unmodified and modified membrane is heterogeneous and does not show observable morphological differences. Pores are also visualized. The light areas correspond to ion exchanger particles and the dark areas to inert polyethylene, which is used as a binder in the membrane manufacturing process [46]. The conducting anion exchanger regions have irregular shapes and are randomly distributed.

A large number of *Aeromonas*, rod-shaped bacteria, with an approximate size of 0.3 µm wide and 1.5 µm long, were detected consistently in the three different zones of the unmodified membranes observed (Figure 5a). Compared to the unmodified membranes, the SEM images of the modified membranes show a lower amount of *Aeromonas* (Figure 5b). These images concur with the static fouling results described in Table 2 and 3, showing a lower adhesion of microorganisms on the modified membranes, which implies that the surface modification with PAA may confer anti-adhesion and/or bactericidal properties to the membranes.

**Figure 6:**
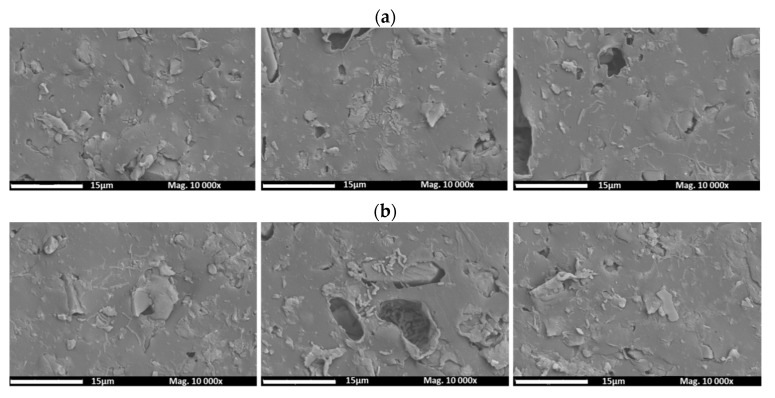
Images of the top surface of the membranes (taken in three different zones) obtained by SEM: (**a**) unmodified membrane and (**b**) modified fouled membrane after chemical cleaning.

An additional test was performed to evaluate the effect of the chemical cleaning procedure (NaClO) proposed on the amount of *Aeromonas* presented on the surface of the unmodified and modified AEMs (Figure 6).

After evaluating a large area of the membranes subject to the cleaning agent, zones with a high density of *Aeromonas* (as can be seen in Figure 6a) were not observed, which demonstrates the efficiency of the proposed cleaning method against biofouling. However, concave regions on the membrane surface may harbor bacteria agglomerates, as can be observed in Figure 6b. The rest of the SEM images for unmodified and modified AEMs fouled with SDS and SDBS (chemical foulants) can be found in the Supplementary Materials (Figures S6 and S7).

## 4. Conclusions

In this work, we report the (bio)fouling characterization of modified and pristine heterogeneous anion exchange membranes, including chemical cleaning studies. The characterization analyses also included FTIR, SEM, and electrochemical tests. The following main conclusions can be derived from the results obtained in this study:

- The presence of poly (acrylic acid) on the membrane surface decreases the water contact angle value of the membranes (improved hydrophilicity). The presence of organic foulants (SDS and SDBS) increased the membrane surface hydrophobicity, while for the *Aeromonas*, this effect was negligible.
- The PAA-modified membranes exhibit a significantly improved anti-adhesion behavior, since a much lower number of total bacteria counts (in the assays with the real water matrices) and *Aeromonas* (in the assays with the fortified bacteria) were attached on its surface.
- A chemical cleaning method using sodium hypochlorite as the cleaning agent demonstrated an effective recovery of the initial membrane properties after (bio)fouling employing natural feedwaters, without compromising the main properties and structure of the samples. As a result, this chemical cleaning strategy can be applied to recover membrane properties and characteristics, such as ion exchange capacity, water contact angle, and membrane conductivity.

Experiments with real matrixes of seawater and river water demonstrated the existence of several types of compounds and microorganisms that may affect the membrane performance. In this regard, the same membrane characterization experiments which were performed with the model solutions must be considered in real systems in future analyses to move forward towards RED implementation in practice.

## Figures and Tables

**Table 1:** Characterization of the surface and sea water used in the static (bio)fouling experiments.

	pH	Total Solids (g/L)	Total Suspended Solids (mg/L)	COD (mg/L O_2_)	Total Bacteria Count (CFU */mL)	Total Coliforms (MPN **/100 mL)	*E. coli* (MPN **/100 mL)
Surface water	7.8	46.5	50.2	220.5	830	750	140
Seawater	8.0	46.8	44.8	60.0	700	270	39

* colony-forming units; ** most probable number.

**Table 2:** Adhesion of microorganisms present at occurrence levels in surface and sea water onto the membrane surface of modified and unmodified membranes.

	Unmodified Membrane			Modified Membrane		
	Total Bacteria Count (CFU/cm^2^)	Total Coliforms (MPN/cm^2^)	*E. coli* (MPN/ cm^2^)	Total Bacteria Count (CFU/cm^2^)	Total Coliforms (MPN/ cm^2^)	*E. coli* (MPN/ cm^2^)
Surface water	1050	<2.5	<2.5	800	<2.5	<2.5
Seawater	275	<2.5	<2.5	38	<2.5	<2.5

**Table 3:** Adhesion of *Aeromonas hydrophila* onto the membrane surface of the modified and unmodified membranes.

	Unmodified Membrane	Modified Membrane
*Aeromonas* cell suspension	5.30 × 10^5^ CFU/cm^2^	1.75 × 10^5^ CFU/cm^2^

**Table 4:** Obtained currents at the maximal applied voltage of 0.6 V.

Membrane Type	Presence/Absence of Foulants	Current (mA)
Unmodified	No fouling	2.687 ± 0.006
	SDS (25 ppm)	2.556 ± 0.018
	SDS (250 ppm)	2.596 ± 0.076
	SDBS (25 ppm)	2.586 ± 0.025
	*Aeromonas* (batch 1)	2.361 ± 0.073
	*Aeromonas* (batch 1) + NaClO cleaning	2.486 ± 0.032
	*Aeromonas* (batch 2)	2.291 ± 0.021
	*Aeromonas* (batch 2) + NaClO cleaning	2.694 ± 0.021
Modified	No fouling	2.946 ± 0.028
	SDS (25 ppm)	2.531 ± 0.028
	SDBS (25 ppm)	2.431 ± 0.040
	*Aeromonas* (batch 1)	2.730 ± 0.031
	*Aeromonas* (batch 1) + NaClO cleaning	2.759 ± 0.031
	*Aeromonas* (batch 2)	2.322 ± 0.045
	*Aeromonas* (batch 2) + NaClO cleaning	2.659 ± 0.042

## Data Availability

Not applicable.

## Supplementary Materials

The following are available online at https://www.mdpi.com/article/10.3390/membranes12070697/s1, Figure S1: Comparison of the lethality of different membrane modifying agents: poly(acryclic) acid (PAA), polyethylenimine (PEI), poly(diallyldimethylammonium chloride (PDDA), poly(dopamine) (PDA), and 3,5-diaminobenzoic acid, Figure S2: FTIR analysis of unmodified (a) and modified (b) membranes after 14 days immersion in river and sea water as well as in a cell suspension (1.2 × 10^8^ CFU/mL) of Aeromonas hydrophila isolated from a real surface water, Figure S3: SEM images before and after 14 days immersion of the unmodified and modified membranes in river and sea water as well as in a cell suspension (1.2 × 10^8^ CFU/mL) of Aeromonas hydrophila isolated from a real surface water, Figure S4: FTIR analysis of unmodified (a) and modified (b) membranes after 7 days immersion in NaOH (4 g/L and 30 g/L) and NaClO (3 mg/L), Figure S5: SEM analysis before and after seven days immersion of the unmodified and modified membranes in NaOH (above: 4 g/L and 30 g/L) and NaClO (below: 3 mg/L), Figure S6: Membranes’ surface images obtained by SEM: (a) unmodified + SDS (25 ppm); (b) unmodified + SDBS (25 ppm); (c) modified + SDS (25 ppm); (d) modified + SDBS (25 ppm); (e) unmodified + SDS (250 ppm), Figure S7: Membranes’ cross section obtained by SEM: (a) virgin unmodified; (b) virgin modified; (c) unmodified + SDS (25 ppm); (d) unmodified + SDBS (25 ppm); (e) modified + SDS (25 ppm); (f) modified + SDBS (25 ppm); (g) unmodified + SDS (250 ppm), Figure S8: Comparison between unmodified and modified commercial Ralex AEMs by means of sulfate rejection. F = feed compartment; R = receiver compartment.
