# Supplementary material for: A Study on Biofouling and Cleaning of Anion Exchange Membranes for Reverse Electrodialysis
Source: Membranes (Basel). 2022 Jul 8;12(7):697. doi: 10.3390/membranes12070697 (PMC9316569; doi:10.3390/membranes12070697)
Supplement: Supplementary file 1 [file membranes-12-00697-s001.zip › membranes-1795621-SI.pdf]

membranes

Supporting Information

A study on biofouling and cleaning of anion exchange membranes for reverse electrodialysis

Gonçalo Tiago [1], Maria Beatriz Cristóvão [1,2], Ana Paula Marques[2], Rosa Huertas[1,2], Ivan Merino-Garcia [3], Vanessa Jorge Pereira[2,4], João Goulão Crespo[1], Svetlozar Velizarov [1]*

[1] LAQV-REQUIMTE, Department of Chemistry, NOVA School of Science and Technology, Universidade NOVA de Lisboa, 2829-516 Caparica, Portugal; g.tiago@campus.fct.unl.pt; jgc@fct.unl.pt; s.velizarov@fct.unl.pt

[2] iBET - Instituto de Biologia Experimental e Tecnológica, Apartado 12, 2780-901, Oeiras, Portugal; b.cristovao@ibet.pt; ana.marques@ibet.pt; rosa.maria.huertaspenela@gmail.com; vanessap@ibet.pt;

[3] Departamento de Ingenierías Química y Biomolecular, Universidad de Cantabria, Avda. Los Castros, s/n, 39005 Santander, Spain; ivan.merino@unican.es

[4] Instituto de Tecnologia Química e Biológica António Xavier, Universidade Nova de Lisboa, Av. da República, 2780-157, Oeiras, Portugal

* Correspondence: s.velizarov@fct.unl.pt;

Toxicity of common modifying agents

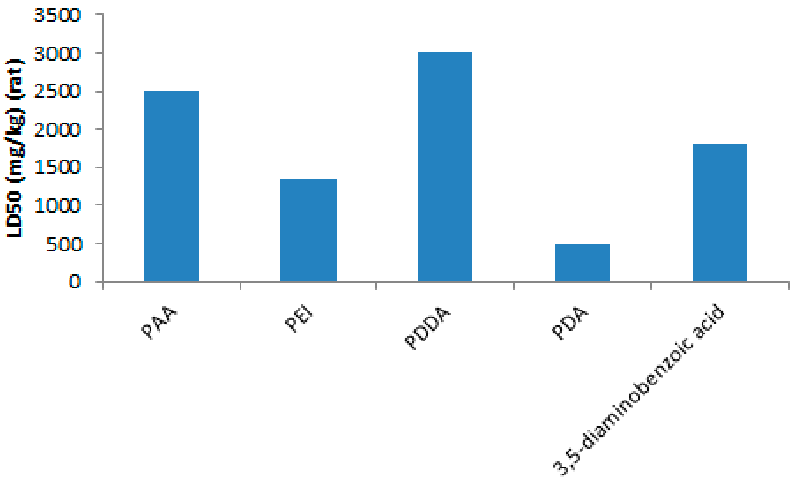

Figure S1. Comparison of the lethality of different membrane modifying agents: poly(acryclic) acid (PAA), polyethylenimine (PEI), poly(diallyldimethylammonium chloride (PDDA), poly(dopamine) (PDA), and 3,5-diaminobenzoic acid.

(Bio)fouling analyses

Static fouling experiments were conducted by immersing the modified and unmodified anion-exchange membranes in river and sea water (characterized in Table 1 of the main manuscript) as well as in a cell suspension (1.2 × 10⁸ CFU/mL) of *Aeromonas hydrophila* isolated from a real surface water. After 14 days, the membranes were analyzed via FTIR (Figure S2) and SEM (Figure S3).

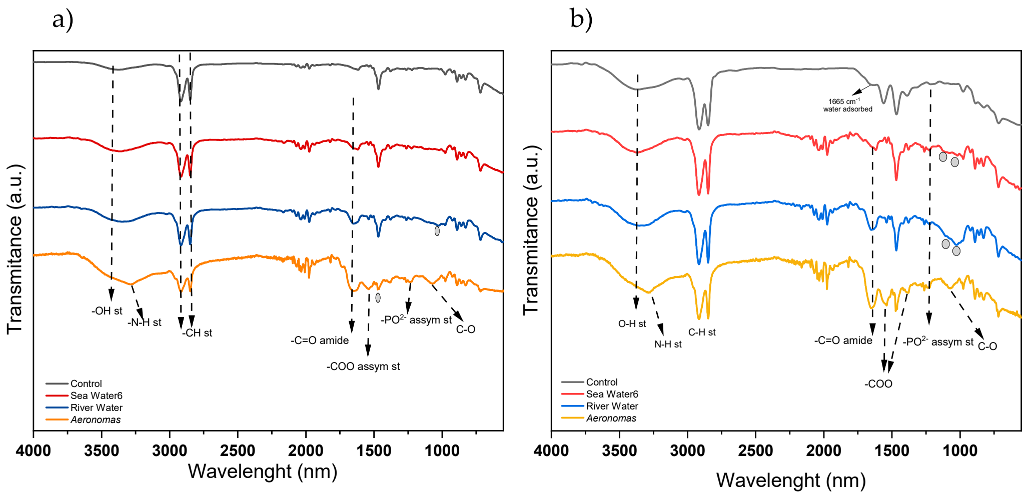

Figure S2. FTIR analysis of unmodified (a) and modified (b) membranes after fourteen-day immersion in river and sea water as well as in a cell suspension (1.2 × 10^8 CFU/mL) of *Aeromonas hydrophila* isolated from a real surface water.

Sea water and river water: notorious differences were not observed between the control and the unmodified as well as modified membranes subject to sea and river water. However, small differences were observed: e.g., a peak around 1652 cm^{-1} for the presence of C=O groups (for sea water and river water) and at around 1029 cm^{-1} that can be associated with C-O groups (river water).

***Aeromonas* biofouling**: Bacteria presence detected-band at around 3283 cm^{-1} that indicates the presence of –N-H groups, at around 1647 cm^{-1} for carbonyl groups for amide groups, as well as 1545 cm^{-1} for the –COO- groups. A decrease in the –CH band at 1464 cm^{-1} was also observed because of the presence of the bacteria on the surface. A new band at around 1229 cm^{-1} was also detected that may be associated to PO2- groups belonging to phospholipids.

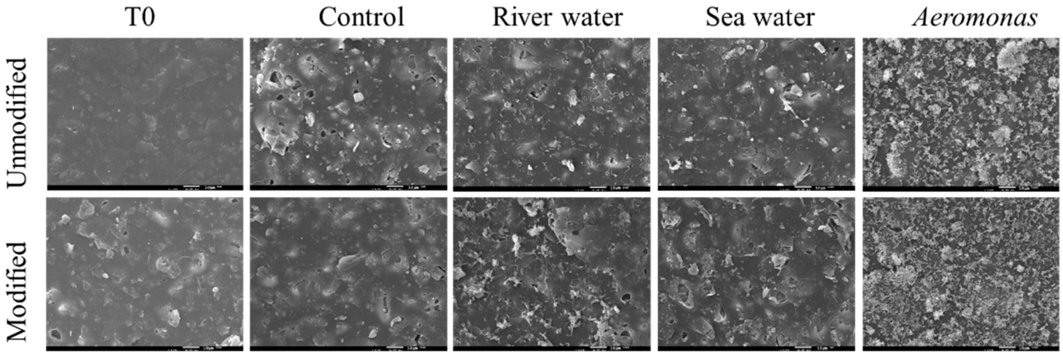

Figure S3. SEM images before and after 14-days immersion of the unmodified and modified membranes in river and sea water as well as in a cell suspension (1.2 × 10^8 CFU/mL) of *Aeromonas hydrophila* isolated from a real surface water.

Effect of chemical cleaning on membrane surface

Coupons of modified and unmodified membranes (2cm x 1cm, Ralex-AEM) were immersed in NaOH (4 g/L and 30 g/L) and NaClO (3 mg/L). After 7 days, the membranes were analyzed via FTIR (Figure S4) and SEM (Figure S5).

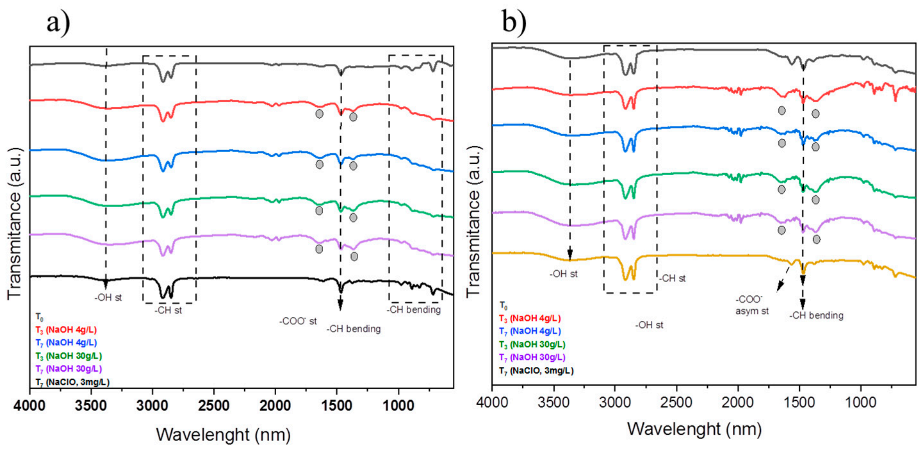

Figure S4. FTIR analysis of unmodified (a) and modified (b) membranes after seven days immersion in NaOH (4 g/L and 30 g/L) and NaClO (3 mg/L).

As represented in Figure S4, the most characteristic bands of the unmodified membrane were the 3384 cm^{-1} for OH groups for water adsorbed, the 2925 cm^{-1}, 2838 cm^{-1} and 1472 cm^{-1} for aliphatic C-H groups as well as the 1624 cm^{-1} for ester groups. Regarding the modified membrane (Figure A8b), in addition to the previous bands, the 1733 cm^{-1} (-C=O st), the 1559 cm^{-1} (-COO- assym st), 1431 cm^{-1} and 1360 cm^{-1} (-COO-sym st) for the carboxylate bond of the ester group formed after modification with the polyacrilyc acid, were also among the most characteristic bands. The water adsorbed can be also presented at 1647 cm^{-1} in accordance with the increasing of the band around 3384 cm^{-1} for hydroxyl groups. After the exposure to NaOH, the FTIR results showed that for both unmodified and modified membranes, new bands appear at approximately 1638 cm^{-1} and 1362 cm^{-1}, which are associated with COO-stretching (asymmetric and symmetric respectively). This could be due to the well-known reaction between the polyester and NaOH. On the other hand, no differences were observed for both membranes after 7 days of exposure to NaClO.

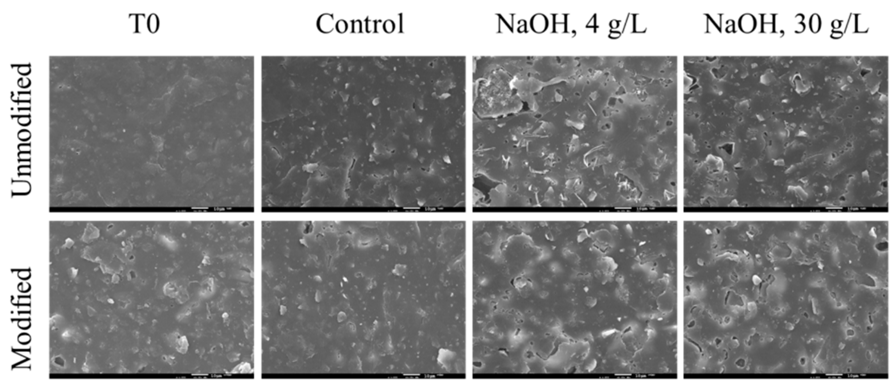

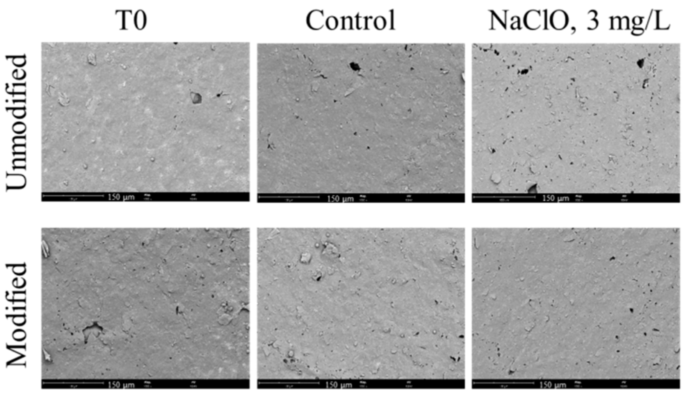

Figure S5. SEM analysis before and after seven days immersion of the unmodified and modified membranes in NaOH (above: 4 g/L and 30 g/L) and NaClO (below: 3 mg/L).

Additional SEM images using model foulants

Figures S6 (membrane surface) and S7 (cross section) display the SEM images of unmodified and modified membranes fouled with model organic solutions of SDS and SDBS.

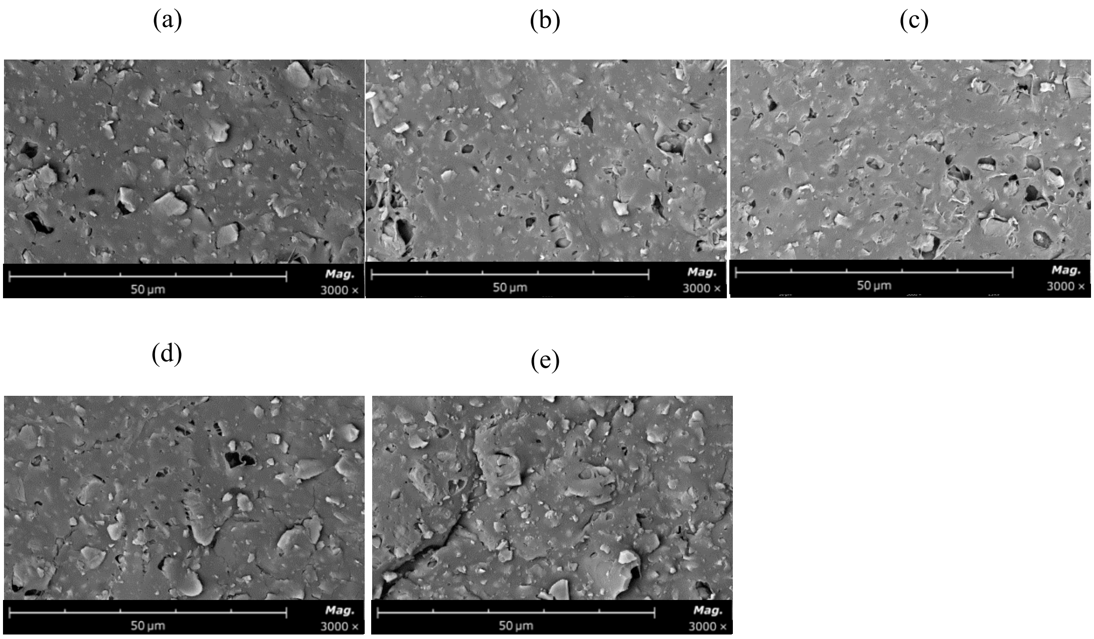

Figure S6. Membranes' surface images obtained by SEM: (a) unmodified + SDS (25 ppm); (b) unmodified + SDBS (25 ppm); (c) modified + SDS (25 ppm); (d) modified + SDBS (25 ppm); (e) unmodified + SDS (250 ppm).

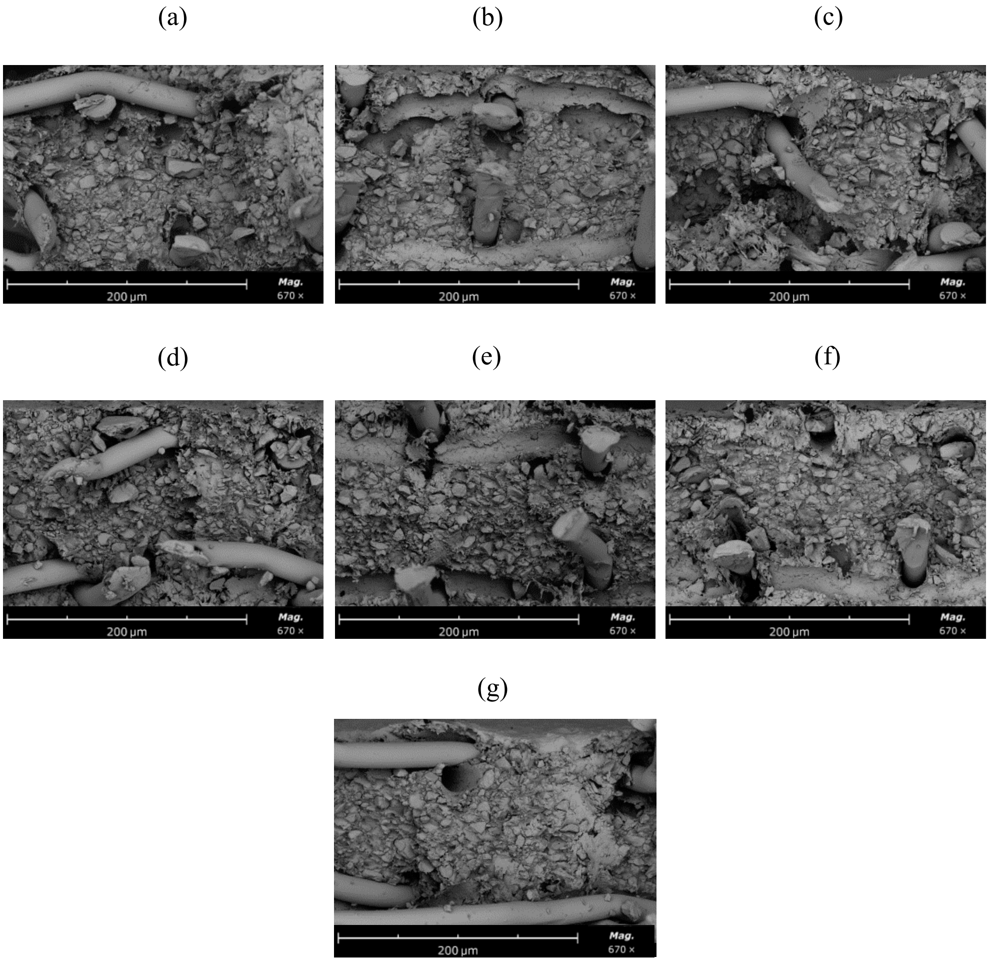

Figure S7. Membranes' cross section obtained by SEM: (a) virgin unmodified; (b) virgin modified; (c) unmodified + SDS (25 ppm); (d) unmodified + SDBS (25 ppm); (e) modified + SDS (25 ppm); (f) modified + SDBS (25 ppm); (g) unmodified + SDS (250 ppm).

A Donnan dialysis test

Figure S8 shows the time evolution of sulfate concentration in the feed and receiver compartment of a two-compartment cell for evaluating the efficacy of the PAA modifying procedure. Since under batch Donnan dialysis conditions, the final state of the system corresponds to the Donnan equilibrium condition the final sulfate concentrations in the receiver compartment were expectedly the same for the unmodified and modified membrane. However, for a continuously operating (meaning mass transport rate-controlled) RED process, the rejection of sulfate would be clearly higher for the modified membrane.

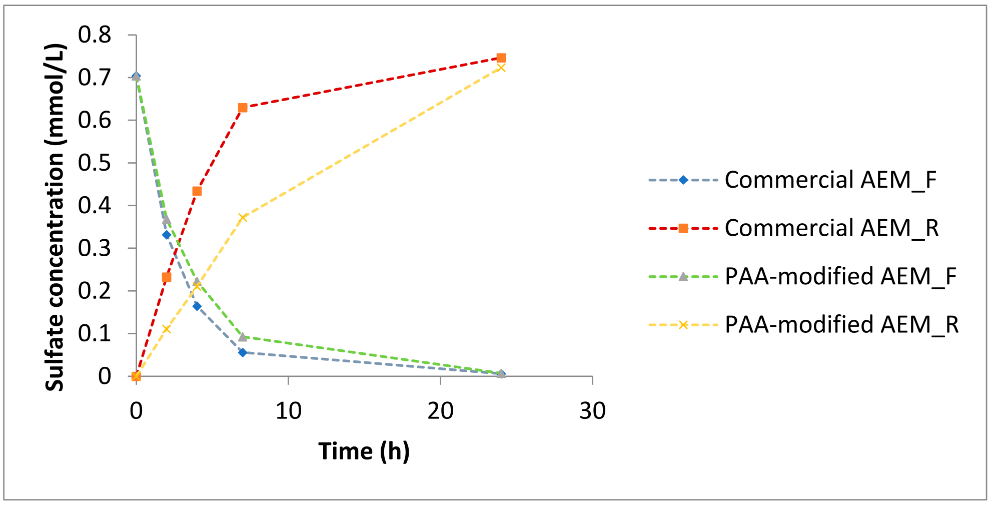

Figure S8. Comparison between unmodified and modified commercial Ralex AEMs by means of sulfate rejection. F = feed compartment; R = receiver compartment.
